# Effect of variable heat transfer coefficient on tissue temperature next to a large vessel during radiofrequency tumor ablation

**DOI:** 10.1186/1475-925X-7-21

**Published:** 2008-07-11

**Authors:** Icaro dos Santos, Dieter Haemmerich, Cleber da Silva Pinheiro, Adson Ferreira da Rocha

**Affiliations:** 1 Department of Electrical Engineering, University of Brasilia, Brasilia, DF 70910-900, Brazil; 2 Division of Pediatric Cardiology, Medical University of South Carolina, 165 Ashley Ave., Charleston, SC 29425, USA; 3 Department of Bioengineering, Clemson University, Clemson, SC 29634, USA

## Abstract

**Background:**

One of the current shortcomings of radiofrequency (RF) tumor ablation is its limited performance in regions close to large blood vessels, resulting in high recurrence rates at these locations. Computer models have been used to determine tissue temperatures during tumor ablation procedures. To simulate large vessels, either constant wall temperature or constant convective heat transfer coefficient (*h*) have been assumed at the vessel surface to simulate convection. However, the actual distribution of the temperature on the vessel wall is non-uniform and time-varying, and this feature makes the convective coefficient variable.

**Methods:**

This paper presents a realistic time-varying model in which *h *is a function of the temperature distribution at the vessel wall. The finite-element method (FEM) was employed in order to model RF hepatic ablation. Two geometrical configurations were investigated. The RF electrode was placed at distances of 1 and 5 mm from a large vessel (10 mm diameter).

**Results:**

When the ablation procedure takes longer than 1–2 min, the attained coagulation zone obtained with both time-varying *h *and constant *h *does not differ significantly. However, for short duration ablation (5–10 s) and when the electrode is 1 mm away from the vessel, the use of constant *h *can lead to errors as high as 20% in the estimation of the coagulation zone.

**Conclusion:**

For tumor ablation procedures typically lasting at least 5 min, this study shows that modeling the heat sink effect of large vessels by applying constant *h *as a boundary condition will yield precise results while reducing computational complexity. However, for other thermal therapies with shorter treatment using a time-varying *h *may be necessary.

## Background

Radiofrequency (RF) tumor ablation is a treatment modality that uses radiofrequency electric current in an attempt to destroy cancer cells by localized heating of tumors. RF ablation is used for liver cancer, with increasing use in other organs such as kidney, lung, bone, and adrenal gland [1].

The heat due to RF heating causes tissue necrosis at predictable temperatures in relatively predictable volumes. During an RF ablation procedure, an active electrode is inserted percutaneously (i.e. through a small incision in the skin), during laparoscopy, or during open surgery with image-guidance into the tumors. Grounding pads are positioned at the patient's thighs or back muscles. RF energy is applied and current flows from the active electrode to the grounding pads. Thus, the patient becomes an element of the electrical circuit.

High RF current densities around the electrode results in resistive heating in surrounding tissue. While it takes several hours to induce cell necrosis at 43°C [2], at 50°C cell death occurs within 2–3 min [3]. The tissue adjacent to the electrode is rapidly heated, and the remainder of the tissue is heated by thermal conduction, which is a slow process. Typically, a single ablation takes 12 – 35 min depending on device type [4]. If the tissue temperature near the electrode is excessive, the tissue desiccates, and this process results in an electrically insulating layer preventing further energy deposition. As a result, the phenomenon limits the volume of the tissue that can be treated. A difference between tumor ablation and hyperthermia treatments (where lower temperatures of 43 – 45°C are used) is that during ablation most of the tissue volume that undergoes necrosis is at temperatures between 50 and 100°C. Therefore, the prevalent effect of cell death is coagulation necrosis conversely to hyperthermia treatments where a number of biologically complex mechanisms are in effect [5].

A shortcoming of current RF ablation devices is the limited performance adjacent to large blood vessels (diameter > 3 mm). When the active electrode is inserted near large vessels, the blood flow drags thermal energy away from the target tissue [6]. This is a heat sink effect that can change both the shape and maximum volume that can be treated. In fact, the distance of the blood vessels from the tumor determines the location of the maximal tissue temperature. As a result, tumors in the vicinities of large vessels are associated with high recurrence rates [7]. A considerable number of mathematical models have been suggested to describe heat transfer between tissue and vasculature [8-20] most of them are not directly applicable to liver tissue due to the specific blood supply of the liver. Most of the blood perfusing the liver (roughly 70%) is venous blood [21]; in addition, there are no counter-current vessels (i.e. venous and arterial vessels adjacent with opposing blood flow) in the liver as present in other tissues, and counter-current vessels are an integral assumption of most heat transfer models. As a result, most computational models of high-temperature tumor ablation simulate microvascular perfusion using the commonly used Pennes formulation [8], and model large vessels separately since the Pennes model does not describe large vessel perfusion accurately, similar to a previous study [22].

In order to estimate the heat sink effect of large vessels, many simulations performed hitherto assigned either constant temperature at the surface of the vessel [6,23] or constant convective heat transfer coefficient, *h*, throughout the RF ablation procedure [24]. In order to estimate the value of *h*, the simulations performed implied that the flow in the vessel is laminar, the thermal boundary layer is fully-developed and *h *remains constant throughout the procedure. Other works support that hypothesis that the flow is laminar in large vessels [25,26]. However, since the heated region during the procedure varies in time and the vessel heated length is small, the thermal boundary layer is not fully-developed. Thus, both the vessel wall temperature and *h *varies during the ablation procedure and cannot be considered constant [27]. Since the magnitude of *h *is a parameter that may significantly impact on the shape and size of the coagulation zone obtained during hepatic RF ablation, it is important to accurately model the time-varying behavior of *h *during the RF ablation of liver tumors in order to correctly determine the coagulation zone size. Thus, the overall objective of this work is to theoretically evaluate the impact of the time-varying *h *on the accuracy of the simulations of RF tumor ablation procedures.

## Methods and models

We used the software FEMLAB (v. 3.2) to generate the finite element model. This software performs coupled electrical-thermal field analysis, and provides all the elements needed to build the model, solve the problem and post-process the results. The heating of tissue during RF ablation is modeled by the bioheat equation (1) [8].

(1) $$\rho c\frac{\partial T}{\partial t}=\nabla \cdot k\nabla T+\vec{J}\cdot \vec{E}-\rho_{bl}c_{bl}w_{bl}(T-T_{bl})+Q_{m}$$

where *ρ*, *c *and *k *are, respectively, the density (kg*m^-3^), the specific heat (J*kg^-1^*K^-1^) and the thermal conductivity (W*m^-1 ^K^-1^) of the liver tissue. $\vec{J}$(A*m^-2^) and $\vec{E}$(V*m^-1^) are current density and the electric field intensity and can be calculated with Laplace's equation, using a quasi-static approximation. *T *is the temperature of the tissue, *T*_*bl *_is the temperature of the blood, *ρ*_*bl *_is the blood density, *c*_*bl *_is specific heat of the blood and *w*_*bl *_is blood perfusion (s^-1^). *Q*_*m*_(W*m^-3^) is the energy generated by metabolic processes and was neglected since it is small if compared to the other terms [28]. Microvascular perfusion was included in the model, considering the Pennes model [8]. Although the perfusion in normal and tumorous tissue can vary greatly, this paper focus on the temperature field close to large vessels and on the behavior of the time-varying *h *during RF ablation rather than simulating a clinical situation. The parameter *w*_*bl *_used in this model was 6.4.10^-3 ^s^-1 ^[29], which is in the range of the perfusion for cirrhotic human liver tissue [30]. Equation 1 states that RF current flowing through the tissue is converted into thermal energy, which in turn cause tissue injury.

The thermal and electrical properties for the various materials of interest are listed in Table 1[6,31-33]. The vessel wall was not modeled separately since it has similar properties as normal liver tissue [34]. Since we are mainly concerned with the impact of *h *on the thermal ablation zone, this study does not model the temperature dependence of the thermal-electrical parameters of the tissue.

**Table 1:** Thermal and electric properties of the materials.

Finite element region	Material	*ρ*(kg*m^-3^)	*c*(J*kg^-1^*K^-1^)	*k*(W*m^-1^*K^-1^)	*σ*(S*m^-1^)
Electrode	Nickel-Titanium	6450	840	18	1.10^8^
Trocar	Stainless steel	21500	132	71	4.10^6^
Insulated trocar	Polyurethane	70	1045	0.026	1.10^-5^
Tissue	Blood	1000	4180	0.543	0.667
Tissue	Liver	1060	3600	0.512	0.333

**Figure 1:**
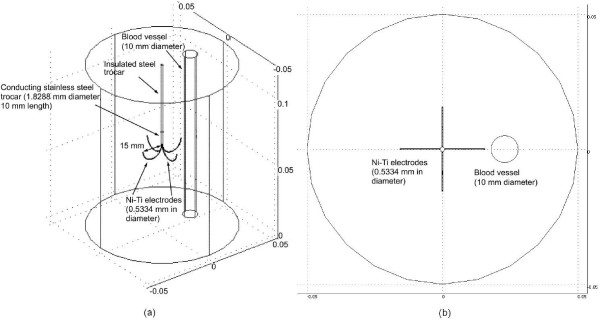
**Model geometry of 3-D FEM.** (a) The probe is completely inserted in the liver and a 10 mm blood vessel is located close to the electrode. (b) Top view of the model.

A three dimensional view of the model and its physical domain are shown in Figure 1(a). Figure 1(b) shows the top view of the model. We set the temperature on the boundary of the model to 37°C and the voltage at outer surfaces at 0 V. We modeled a multi-tine electrode (15 gauge, model-30, Rita Medical Systems, Inc., Fremont, CA).

**Figure 2:**
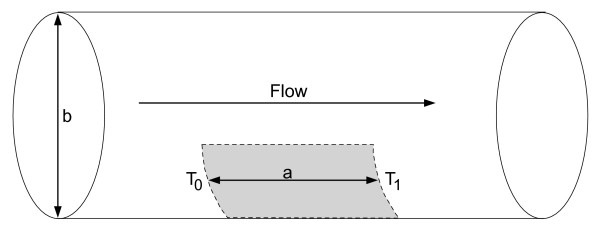
**Model used in the analysis of boundary conditions of the blood vessel.** The gray area represents the heated length.

The hepatic tissue where the electrode is embedded was modeled as a cylinder (100 mm diameter × 120 mm length). The initial temperature of tissue-electrode system and of the boundary of the model was set to 37°C. The voltages on the outer surfaces of the model were 0 V, assuming the Dirichlet boundary condition. The convective boundary condition was considered in the tissue-vessel interface. Thus, the heat sink effect of the blood flow inside the vessel was modeled by a convective boundary condition at the tissue-vessel interface. The value of convective coefficient was set in the model according to Equation 2, which assumes that the blood behaves as a Newtonian fluid in large vessels, the flow is laminar and the geometry of the vessel is linear [27]. Subsequently, with the use of Equation 2, the value of the convective coefficient is updated at each time step and is set as the new convective boundary condition on the vessel wall for the next step.

(2) $$h=\frac{\rho cu(T_{1}-T_{0})b}{[0.25(2T_{s}+T_{1}+T_{0})-T_{m}]a},$$

where *h *is the convective heat transfer coefficient (W*m^-2 ^*K^-1^) and accounts for the large vessel perfusion, *ρ *is the density of the blood (kg*m^-3^), *c *is the specific heat of the blood (J*kg^-1 ^*K^-1^), *T*_0 _is the inlet blood temperature (37°C), *T*_1 _is the temperature at the vessel outlet and *T*_*s *_is the maximum temperature of the heated region at the vessel wall. The parameters *a *and *b *are, respectively, the length of the heated region and the diameter of the vessel (Figure 2). *u *is the constant mean value of the blood velocity (0.2 m*s^-1^), considering the blood flow with laminar profile. Also, *T*_*m *_is 0:5·(*T*_0 _+ *T*_1_). The temperatures *T*_0 _and *T*_1 _are set at 37 and 37.05°C when the temperature of the vessel wall is 50°C. This choice is based on both theoretical and experimental work performed by our group [27,35]. For other vessel wall temperatures, we used linear interpolation to evaluate the temperature *T*_1_. The temperature *T*_0 _is kept constant at 37°*C*. The length of the heated region was calculated based on regions where the temperature in the vessel is above 37.01°C. Equation 2 considers the blood as a Newtonian fluid and one might be cautious about extending this equation to vessels smaller than 0.5 mm in diameter [27].

Initially, the profile temperature is uniform and there is no heated region in the vessel. Thus, the convective heat transfer coefficient is set initially at 0 W*m^-2 ^*K^-1^. Temperature controlled RF ablations were simulated by varying the voltage applied to the electrodes during the procedure. The voltage was controlled in order to keep the maximum tissue temperature at 90°C. We performed temperature control by means of a proportional-integral controller as described previously [36]. The control algorithm for the finite element model is described by equations 3 and 4.

(3) $$V=K_{p}e+K_{i}\int_{0}^{t}e(t)dt$$

(4) *e *= *T*_*s *_- *T*_*t*_

where *T*_*t *_is the electrode tip temperature, *T*_*s *_is the set temperature measured at the tip of the electrode where maximum temperatures occur, and *K*_*p *_and *K*_*i *_are control parameters of the PI controller. The control parameters determine the behavior of the algorithm, e.g., response time, overshoot, swinging. The parameters we used for the PI controller were *K*_*p *_= 0:4 and *K*_*i *_= 0:04. These values were obtained based on tests performed with the dynamic system. In order to implement the time integral of the temperature difference between the electrode tip and the set temperature (equations 3 and 4), we added an ordinary differential equation in Femlab.

In this study, the maximum hepatic tissue temperature was kept at 90°C and the maximum power applied during the procedure was 28 W. We used the 50°C-isotherm to determine the coagulation zone boundary as has been done in previous models of RF tumor ablation [6,24,28,37]. We used the temperature at the tip of the electrode for convergence test. We refined the mesh and run the analysis again. We searched the optimal mesh size in order to keep the simulation fast while keeping the accuracy better than 0.01°C at the tip of the electrode. We tested 2 mesh sizes: 65,759 and 13,714. We concluded that the 13,714-element model satisfied our requirement. The time step size was chosen at 0.05 s, so that the maximum temperature change was smaller than 0.01°C during each step. The simulations were performed on a PC with a 2.4 GHz PENTIUM Celeron CPU, with 1 GB of RAM and 30 GB of hard disk space. For post processing, we employed the built-in module in FEMLAB and MATLAB. The computation time required for 10 min simulation was about 3 hours.

We placed the electrode in two different configurations: (a) 1 mm away from the vessel (shown in Figure 1) and (b) 5 mm away from the vessel. For each of these two configurations, we performed three studies.

• The behavior of the convective heat coefficient on the vessel;

• The evolution of the coagulation zone volume when the time-varying behavior of *h *is taken into account and when *h *is constant and equal to the value of the time-varying *h *at the end of the 10 minutes ablation;

• The behavior of the maximum temperature at the vessel wall with both constant and time-varying *h*.

## Results

### The behavior of the convective heat coefficient at the vessel wall

**Figure 3:**
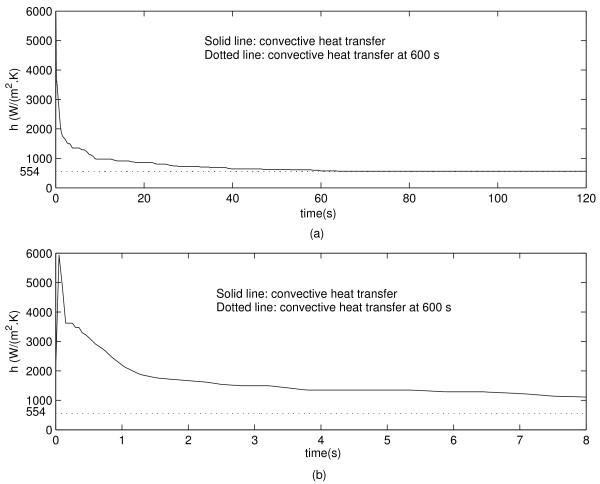
**(a) Convective heat transfer coefficient during 120 s simulation for the electrode 1 mm away from the vessel.** (b) Close up view for the first 8 s of simulation. The dotted line indicates the steady state value of *h*.

Figure 3(a) shows the evolution of the convective heat transfer coefficient at the vessel wall when the electrode is placed 1 mm away from the vessel. One can see in Figure 3(b) that *h *increases sharply at the beginning of the ablation procedure, followed by a sharp decrease within a second. The final value of *h *is indicated with a dotted line in Figure 3.

**Figure 4:**
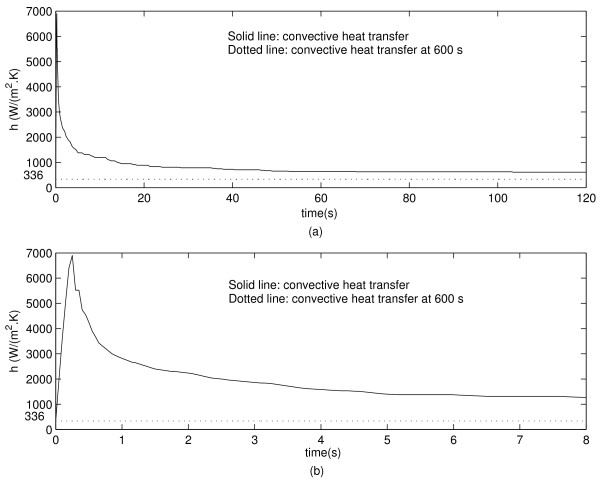
**(a) Convective heat transfer during 120 s simulation for the electrode 5 mm away from the vessel. **(b) Zoom for the first 8 s of simulation.

Figure 4(a) shows the evolution of the convective heat transfer coefficient on the vessel wall when the electrode is located 5 mm away from the vessel. Figure 4(b) shows that the maximum value of *h *occurs approximately at 0.5 s and decreases sharply thereafter. The final value of *h *is shown in Figure 4(a) and 4(b).

### The evolution of the coagulation zone volume for varying *h *and for constant *h *cases

**Figure 5:**
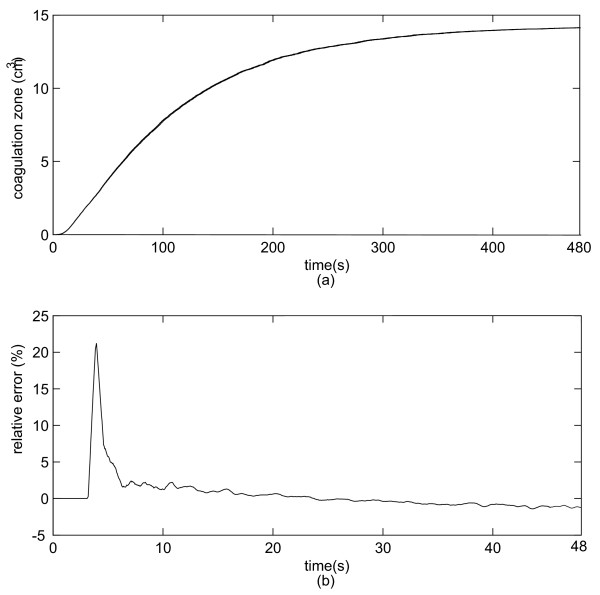
**(a) Coagulation zone volume evolution for varying *h *and constant *h *for the electrode 1 mm away from the vessel. **(b) Relative error (%) between the two configurations. Notice that the two curves are so close that they are visually indistinguishable and that the curves have different time scale.

We analyzed the evolution of the coagulation zone volume (temperature greater than 50°C). First, we analyzed the coagulation zone development when varying *h *was taken into account. Second, we considered *h *as constant and equal to the final value of *h *in the first simulation. Figure 5(a) shows the coagulation zone volume development for both constant and time-varying *h *when the electrode is 1 mm away from the vessel. The absolute error is very small so that the curves are almost identical. Figure 5(b) shows the relative error (%) between the results obtained with the constant *h *and time-varying *h*. We also simulated the volume coagulation zone when the electrode is 5 mm away from the vessel. However, the differences in coagulation zones for constant *h *and time-varying *h *were below to 0.5% and therefore considered negligible.

### The behavior of the maximum temperature at the vessel wall for varying *h *and for constant *h*

**Figure 6:**
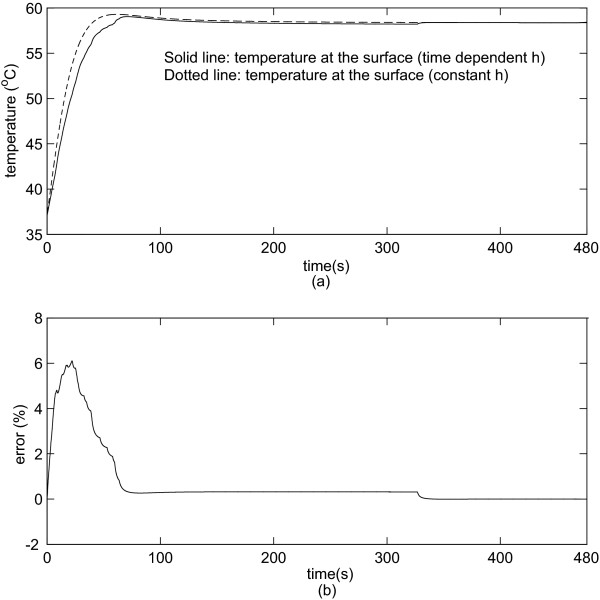
**(a) Maximum temperature at the surface of the vessel (1 mm from the electrode).** (b) Relative error (%) using constant *h *and varying *h*.

We analyzed the evolution of the temperature at the surface of the vessel. We determined the maximum temperature at the surface when the distance between the electrode and the vessel is 1 and 5 mm. Figure 6(a) shows the maximum temperature at the vessel wall when the electrode is 1 mm away from the vessel. Figure 6(b) shows the relative error (%). Figure 6(a) and 6(b) reveals that the difference in temperature is negligible after 80 s since the relative error approaches to 0%.

**Figure 7:**
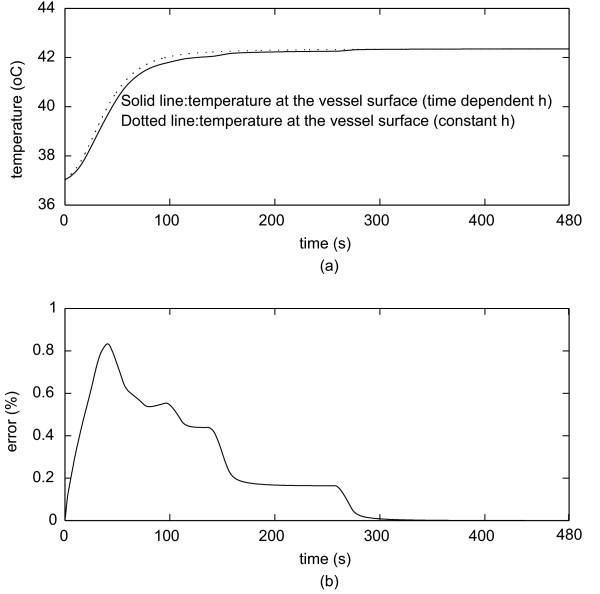
**(a) Maximum temperature at the surface (5 mm from the electrode).**(b) Percentage error using constant *h *and time-varying *h*.

Figure 7(a) shows the temperature when the electrode is 5 mm from the vessel. Figure 7(b) shows the percentage error. One can see that the difference in temperature is nearly negligible during the whole procedure.

## Discussion

The aim of this study was to evaluate the impact of the time-varying behavior of the convective heat transfer coefficient *h *for the determination of the coagulation zone during RF tumor ablation procedures. We have simulated two different behaviors of the convective heat transfer coefficient: constant and variable. We also studied two configurations with the electrode either 1 mm, or 5 mm distant from a large vessel 10 mm in diameter [38]. When the electrode is 1 mm away from the vessel, *h *initially rapidly increases up to 6000 W*m^-2 ^*K^-1 ^followed by a sharp decrease to approximately 554 W*m^-2 ^*K^-1 ^within 60 s (Figure 3). When the electrode is 5 mm away from the vessel, the convective coefficient rapidly increases to approximately 7000 W*m^-2 ^*K^-1 ^and then rapidly decreases to 336 W*m^-2 ^*K^-1 ^(Figure 4). This behavior occurs because the thermal energy reaches the vessel rapidly due to close proximity to the electrode. Thus, *h *initially increases due to the increase in temperature on the vessel wall, and after a while the temperature increase extends along the vessel and the convective heat transfer coefficient decreases. Hence, the maximum value of *h *for the first case occurs around 0.2 s, and for the second case it occurs around 0.5 s after start of the ablation. This behavior becomes more obvious by observing Equation 2. Initially, the vessel wall is not heated yet, and *h *is 0 W*m^-2 ^*K^-1 ^because there is no heat transfer to the vessel. Then the temperature at the vessel starts to increase and also the heated length grows larger typically up to 11 cm. Initially the heated length is very small compared to the temperature difference *T*_1 _- *T*_0_, thus *h *sharply increases. After a few seconds, the length starts to increase faster than the difference (*T*_1 _- *T*_0_) and *h *starts to decrease until both (*T*_1 _- *T*_0_) and *h *show little change because equilibrium between heat loss and heating is reached. Recall that the thermal boundary layer is very small at the entrance region. Thus, *h *is very high [39]. It is noteworthy that if one wants to measure the dynamic behavior of the heat convection coefficient, the instrumentation must have a fast dynamic response and a very high static range as one can see from figures 3 and 4. We also analyzed the coagulation zone for the two cases of time-varying *h *and constant *h*. In the first few seconds and when the electrode is close to the vessel, the time-varying *h *has considerably higher values than the constant *h *and the relative error in coagulation zone volume due to the assumption of a constant *h *is very high (22%). For RF liver ablation this may not be important since initially the ablation volume is small (with a small absolute error), and the volume near the beginning of the treatment is not of high clinical value. As the time increases, the time-varying *h *sharply decreases, approaching its final value after 80 s for the 1 mm case and after 120 s for 5 mm case. Thus, the final coagulation zone volume is nearly identical in both cases. We also investigated the maximum temperature at the wall of the vessel, where tumor recurrence is possible due to insufficient temperatures. The maximum error in temperature is around 6% for the first 50–100 s, but then again is close to zero at the end of the ablation. Consequently, for tumor ablation procedures typically lasting at least 5 min, a very important result is the fact that modeling the heat sink effect of large vessels by applying constant *h *as a boundary condition will yield accurate results for RF ablation while reducing computational complexity. For other thermal therapies with shorter treatment times (e.g. 45 – 60 s long cardiac ablation close to coronary vessels) using a time-varying *h *might be required but needs to be further investigated.

## Conclusion

Previous studies considered a constant heat transfer coefficient throughout the ablation procedure. In this work, simulations were performed using a more realistic, time-varying analytical expression of the convective heat transfer coefficient, which depends on the blood velocity and on the temperature distribution on the vessel wall. The simulations showed that the assumption of a constant convective coefficient leads to precise results when it is used for typical ablation procedures. Only during the first 1–2 min, a time-varying coefficient produces noticeable different results. However, this has no clinical impact for RF liver ablation procedure, which typically takes over 5 min.

## Competing interests

The authors declare that they have no competing interests.

## Authors' contributions

IS and AFR conceived the study; IS and CSP carried out the computer simulation; AFR and DH helped with the interpretation of the results and with the draft of the manuscript. All authors have read and approved the final manuscript.
